# Prevalence and determinants of RH alloimmunization in Rh-negative women in teaching hospitals of Addis Ababa, Ethiopia: a hospital-based cross-sectional study

**DOI:** 10.3389/fgwh.2023.1167736

**Published:** 2023-08-14

**Authors:** Melat B. Maruta, Kiflom Tesfaye, Esayas Birhanu, Nuradin Yigazu, Mohammed Yuya, Adera Debella, Ibsa Mussa

**Affiliations:** ^1^ Obstetrics and Gynecology, Menelik Comprehensive Specialized Hospital, Addis Ababa, Ethiopia; ^2^ Obstetrics and Gynecology, College of Health Sciences, Addis Ababa University, Addis Ababa, Ethiopia; ^3^ Dader General Hospital, Dader, Ethiopia; ^4^ School of Public Health, College of Health and Medical Science, Haramaya University, Harar, Ethiopia; ^5^ School of Nursing and Midwifery, College of Health and Medical Science, Haramaya University, Harar, Ethiopia

**Keywords:** prevalence, RH, alloimmunization, associated factors, Rh-D sensitization, Addis Ababa, Ethiopia

## Abstract

**Background:**

Despite the implementation of immunization with an anti-D antigen for pregnant women, adverse pregnancy outcomes continue to occur in Ethiopia and most Sub-Saharan African countries. Consequently, the woman's obstetric care is compromised, and there is an increase in perinatal morbidity and mortality. In Ethiopia, the burden of the disease is not well understood, and no research has been conducted in the study area. Therefore, this study aims to determine the prevalence and determinants of Rh alloimmunization in Rh-negative women receiving care at Addis Ababa teaching hospitals.

**Methods:**

An institutional-based cross-sectional study was conducted from 5 October 2020 to 5 May 2021, among 328 Rh-negative pregnant women who received antenatal care and delivery services at Teaching Hospitals under Addis Ababa University. Face-to-face interviews were used to gather data using a pre-tested structured questionnaire, and a chart review was performed using a checklist. The data were entered into Epidata version 3.1 and analyzed using SPSS version 22. Multivariable analysis and logistic regression were used to evaluate the predictors, and the results were presented as an adjusted odds ratio (AOR) with a 95% confidence interval. Statistical significance was declared at a *p*-value < 0.05.

**Results:**

Among Rh-D negative individuals, 56(17.1%) were alloimunized with 95% CI (15.1%, 19.23%). The prevalence of Rh-D negative was 2.1% with 95% CI (1.56%, 2.76%). Factors such as unemployment [AOR = 2.28, 95% CI: 1.21, 4.28], failure to use anti-D prophylaxis in previous pregnancy [AOR = 2.08, 95% CI: 1.10, 3.92), and the presence of sensitizing events [AOR = 0.52, 95% CI: 0.27, 0.84] were statistically significant with the outcome variables.

**Conclusions:**

This study pointed out that the prevalence of Rh was relatively large and that almost one in every five pregnant women was alloimunized. Factors such as unemployment and failure to use anti-D prophylaxis in a previous pregnancy were found to be associated with outcome variables. Therefore, all stakeholders and concerned entities should prioritize enhancing access and affordability to anti-D prophylaxis to prevent the occurrence of Rh alloimmunization and its associated adverse outcomes.

## Introduction

The Rhesus blood group system is the second most clinically significant blood group system, following the ABO blood group (1). The Rhesus antigen is a protein found on the surface of the membrane of human red blood cells. Isoimmunization occurs when a person is immunized with an antigen derived from a similar subject if the antigen was previously absent (2, 3). If a mother is Rh-D negative and the fetus is Rh-D positive, she may develop antibodies if exposed to fetal antigens from the Rh-D positive fetus and this process labeled to be as Rh-D sensitization (2, 4–7).

The study pointed out that the prevalence of Rh-D antigen varies significantly across different races, with a higher prevalence in Africans and a lower prevalence in Asians (8). Transplacental or feto-maternal hemorrhage (FMH), which occurs during medical or therapeutic termination of pregnancy and illegal abortion, ectopic pregnancy, miscarriage, or birth results in sensitization to the D antigen in the case of different RH types in the mother and the fetus (8–10). Once it has happened, sensitization cannot be reversed. This sensitization may cause the mother to develop an immunological memory for future pregnancies. Rhesus incompatibility during pregnancy is a major cause of perinatal morbidity and mortality (9, 11, 12).

In Ethiopia, alloimmunization prevention is inadequate and, in some cases, nonexistent following potentially sensitizing events during the medical termination of pregnancy in Rh-negative women. Due to a lack of data management, information about previous pregnancies and pregnancy terminations is frequently missing in patients' medical notes (13). Despite the fact that the prevalence of the Rh-D negative phenotype is significantly lower among Africans than among Caucasians, Rh alloimmunization remains a major cause of perinatal morbidity in Ethiopia and has resulted in a woman's obstetric care being jeopardized due to the unavailability of anti-D immunoglobulin (14).

Despite the fact that the risk of sensitization is largely determined by the extent of the maternal immune response, the volume of transplacental hemorrhage, and the presence of ABO incompatibility, there is an urgent need in Ethiopia for the implementation of universal access to anti-D immunoglobulin for the Rh-negative pregnant population (15–17). In cases of potentially sensitizing events such as amniocentesis, cordocentesis, antepartum hemorrhage, vaginal bleeding during pregnancy, external cephalic version, abdominal trauma, intrauterine death and stillbirth, inutero therapeutic interventions, miscarriage, and therapeutic termination of pregnancy, anti-D immunoglobulin is needed (18, 19). To prevent sensitization, all D-negative women who gave birth to Rh-D-positive babies should receive at least a single 300 mcg or 1,500 IU dose of RhIG within 72 h after delivery. A maternal sample should also be taken roughly 1 h after birth and tested for FMH in excess of 30 ml of fetal blood. If RhIG is not adequately provided, roughly 17% of Rh-D-negative mothers who give birth to Rh-D-positive babies become alloimmunized. RhIG prophylaxis decreased the overall risk of Rh immunization from 13.2 to 0.2% (4, 20), and testing for large FMoH reduced the risk even further to 0.14% (3, 21). As a result, Rh-D immunization may be further reduced by strict adherence to guidelines for determining FMH and adjusting RhIG accordingly, or by the routine administration of extra RhIG following a non-spontaneous delivery and/or a complicated or prolonged third stage of labour (4, 5).

Despite the commitment of various stakeholders and the government, the prevalence and determinants of alloimmunization remain unabated. Moreover, as far as the knowledge of investigators is concerned, there is a paucity of documented evidence regarding the problems under study, generally at the county level and particularly at the study area level. Thus, the purpose of this study was to assess the prevalence and determinants of Rh alloimmunization among Rh-negative pregnant women in the teaching hospital of Addis Ababa.

## Methods and materials

### Study design, setting, and period

An institutional-based cross-sectional study was conducted from 5 October 2020 to 5 May 2021 among 328 Rh-negative pregnant women who came to the antenatal care and delivery services of Teaching Hospitals in Addis Ababa, Ethiopia. It was conducted at three referral teaching hospitals, namely Tikur Anbesa Specialized Hospital, Gandhi Memorial Hospital, and Zewditu Memorial Hospitals. These hospitals serve as central referral teaching hospitals, and all obstetric emergencies, including high-risk pregnancies, are referred to these hospitals from Addis Ababa and its vicinity.

### Population and eligibility criteria

All pregnant women who visited the three teaching hospitals during the study period were considered the source population, whereas all Rh-negative pregnant women were the study population. Women who were critically ill and unable to provide the required information during data collection were excluded from the study.

### Sample size determination and sampling procedures

In this study, the maximum required sample size was calculated using the single population proportion formula by considering the following assumptions. Taking the prevalence of RH alloimmunization (*p* = 0.5), 50% of P, and the fact that there is no study conducted in the area, a 95% confidence level (Z*α*/2 = 1.96), to increase the representativeness of the sample size and boost the precision, 5% (*α* = 0.05) was considered a tolerable margin of error. Thus, the final sample size became 384.

The total number of Rh-negative pregnant women who attended antenatal and delivery care were considered the study population. As a result, all pregnant women who were RhD-negative and met the inclusion criteria were included in the study, which comprised 328 in this case.

To begin the study, Addis Ababa University-affiliated facilities, notably Tikur Anbesa Specialized Hospital, Gandhi Memorial Hospital, and Zewditu Memorial facilities, were chosen based on the total number of client flows. Based on the population size of the three health institutions, a sample size of 384 was assigned to each facility. The total sample size required and the total number of mothers expected to come for ANC and delivery in each health facility over a seven-month period were divided by the sum of the total number of mothers expected to visit ANC and delivery services in the three health facilities to calculate the sample size to be allocated to each hospital. Then, all 9,876 pregnant women who came for ANC services and all 6,018 pregnant women who came for delivery services, which totaled 15,894 study participants, consented and accepted to undergo a laboratory test to rule out whether they were RH negative or positive. Then, we took all Rh-D-negative women and determined the status of alloimmunization.

### Data collection methods

An interviewer-administered, pretested structured questionnaire was used to collect data on socio-demographic variables such as age, marital status, educational status, occupation, and religion of the participants (3, 8, 14, 20, 22). The wording and sequence of questions were designed in such a way that the sequence of ideas (from general to specific and from easy to difficult questions) was maintained. The data relating to mothers were taken during the ANC service utilization and for women during the intrapartum period while the mother was relatively stable. Twelve trained laboratory technicians were used to collect data, and the task was closely supervised by the investigators. First, we translated it while keeping the purpose of the questionnaire and the intent of the questions in mind. It was translated by group members who were bilingual experts. To ensure the translation's accuracy, the questionnaire was translated back into English by someone who had not seen the original version and was unfamiliar with the questionnaire's context. The back-translated version was then compared to the original, and any meaning differences were corrected. After that, to ensure cross-validity, we tried to interview a set of respondents in English and another set in a local language such as Amharic; their answers were then compared to detect differences in understanding. Finally, pretesting was conducted to identify questions that were poorly understood, ambiguous, or elicited hostile or other undesirable responses. We attempted to conduct a pretest using the already-translated questionnaire. We tried to implement all the steps in pretesting, such as obtaining an evaluation of a questionnaire and testing the revised questionnaire through its paces on friends, colleagues, and so on. Statistically, we performed Cronbach's Alpha, which is a measure used to assess the quality of our employed instruments. The result was 0.89, which was within acceptable ranges. A laboratory technician determined the ABO and Rh blood groups using the slide method. Each study participant had a drop of blood (approx. 40 µl) placed in three different locations on a glass slide. Tulip Diagnostics Private Limited's *in vitro* diagnostic reagents were used for monoclonal blood group antibodies. Using an applicator stick, a drop of each antiserum, A, B, and D, was added to and mixed with each blood drop. The mixture was then gently rocked for 2 min to check for agglutination. The results of agglutination were recorded immediately after waiting for two minutes. Agglutination in blood drop A was assigned to group A, and agglutination in blood drop B was assigned to group B. The agglutination in both drops was considered group AB, and if both blood drops were not agglutinating, it was considered group O. Agglutination in blood drop D was considered Rh-D positive, whereas no agglutination was considered Rh-D negative.

### Variables and their measurement

**Rh factor** is an inherited protein found on the surface of red blood cells. If the blood has the protein called Rh positive but doesn't contain the protein called RH negative (8, 21).

**Rh Alloimmunization** occurs when the maternal immune system is sensitized to Rh D erythrocyte surface antigen (8, 21).

**Coombs' Tests** are tests done to find certain antibodies that attack red blood cells (8, 21).

**Indirect Coombs' tests** find certain antibodies that are in the liquid part of the blood. These antibodies can attack RBCs (8, 21).

**Direct Coombs' tests** are used to detect antibodies or complement proteins attached to the surface of RBCs (8, 21).

### Data quality control

The questionnaire was initially prepared in English and then translated into the local languages by a bilingual expert in Amharic. Then, it was translated back into an English version to ensure its consistency. The data collectors and supervisor received training on the data collection tool and procedures. Before the actual study data collection, a pretest was conducted among 5% of the study participants in similar settings. The investigators and experienced research supervisors provided regular supervision.

### Data processing and analysis

The collected data were first examined for consistency and completeness. They were then purified, coded, and added to EpiData version 3.1 for additional analysis. For analysis, the entered data were exported to SPSS version 22. Frequency tables and figures were used to perform and publish descriptive and summary statistics. In order to look for a relationship between the independent factors and the outcome variable, a binary logistic regression model was built. By using Hosmer-Lemeshow statistics and Omnibus tests, the model's fitness was evaluated.

The actual determinants of the outcome variables were found using multivariable analysis. Using the standard error and co-linearity statistics, a multi-collinearity test was performed to determine whether there was any association between the independent variables, and no evidence of collinearity effects was found. The VIF (Variance Inflation Factor) was 0.951. To determine the direction and degree of the statistical link, the odds ratio (OR) and the 95% confidence interval (CI) were used. A *p*-value of 0.05 was used to denote statistical significance in all univariate and multivariable analyses.

## Results

### Socio-economic and demographic characteristics

A total of 328 study participants aged between 15 and 49 years were involved, yielding a response rate of 100%. The mean (±SD) age of the study participants was 26.6 (±6.157). The majority, 300 (91.5%) of the research participants, were married, and 133 (40.6%) had only completed primary school. Among the 328 study participants, half (50.6%) were employed. The Amhara ethnicity comprised 112 (34.1%) participants,, and 201 (61.3%) were Orthodox (Table 1).

**Table 1:** Socio-demographic characteristics of the study participants in TASH, GMH, and ZMH, Addis Ababa, Ethiopia, 2021.

Variables	Categories	Frequency	Percentage
Age	15–19	3	1.1
	20–24	91	27.7
	25–29	140	42.6
	30–34	69	21
	>/=35	25	7.6
Ethnicity	Oromo	96	29.3
	Amhara	112	34.1
	Tigre	40	12.2
	Gurage	52	15.9
	Others^a^	28	8.5
Religion	Orthodox	201	61.3
	Muslim	74	22.6
	Protestant	47	14.3
	Others^b^	6	1.8
Current marital status	Single	20	6.1
	Married	300	91.5
	Other^c^	8	2.4
Education	Unable to read and write	19	5.8
	Primary	133	40.6
	secondary	109	33.2
	Collage and above	67	20.4
Occupational status	Unemployed	162	49.4
	Employed	166	50.6

^a^ Silte, Somali.

^b^ Wakefeta, Catholic.

^c^ Separated, divorced, widowed.

### Blood group profile of study participants

**Figure 1:**
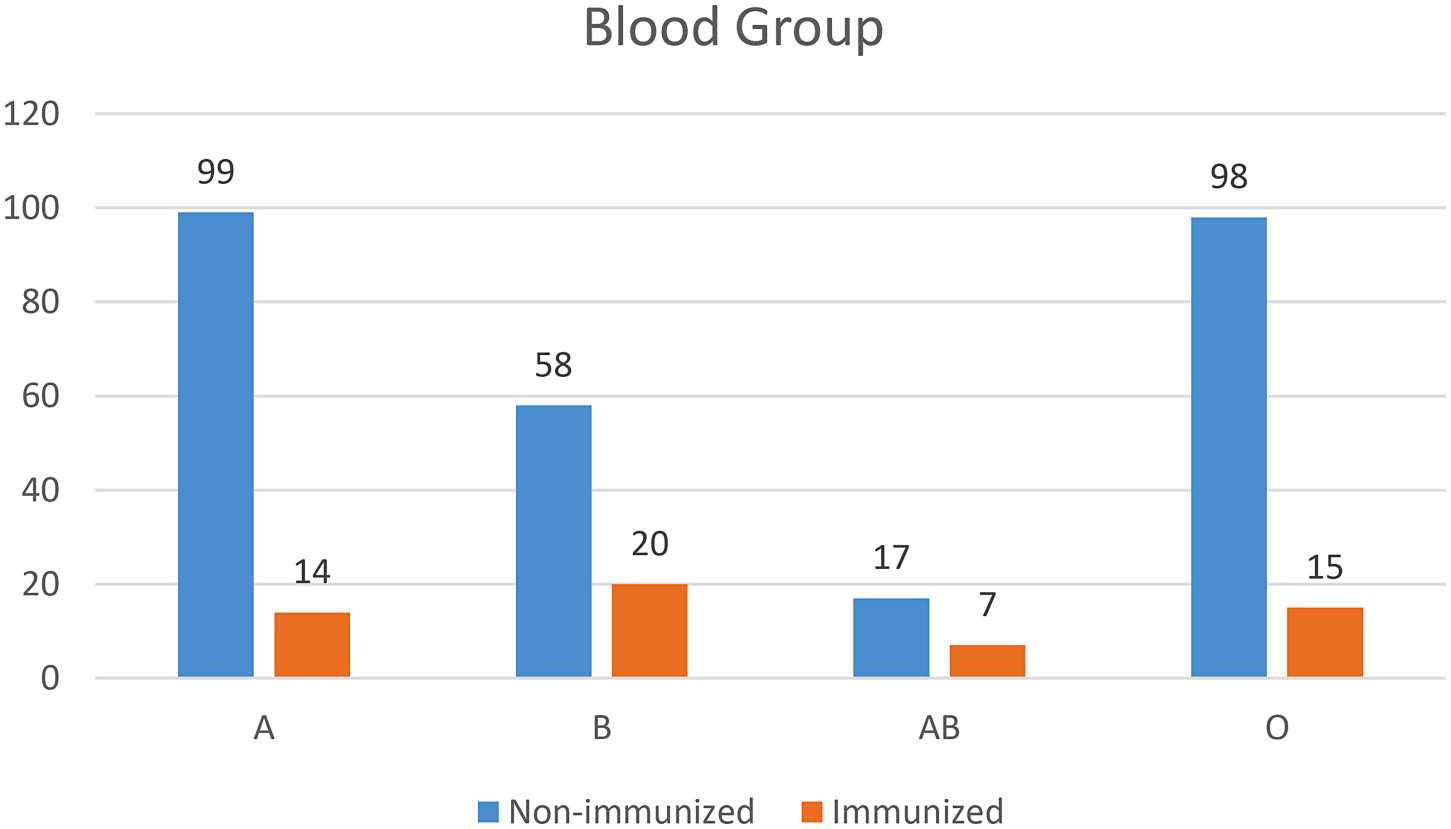
Blood group profile of pregnant women who came to ANC and delivery services in teaching hospitals of Addis Ababa, Ethiopia, 2021.

As shown in the figure below, 113 (34.5%) of the 328 study participants were A and O blood groups, while 24 (7.3%) were AB blood groups (Figure 1).

### Prevalence of Rh-D negative and Rh alloimmunization

**Figure 2:**
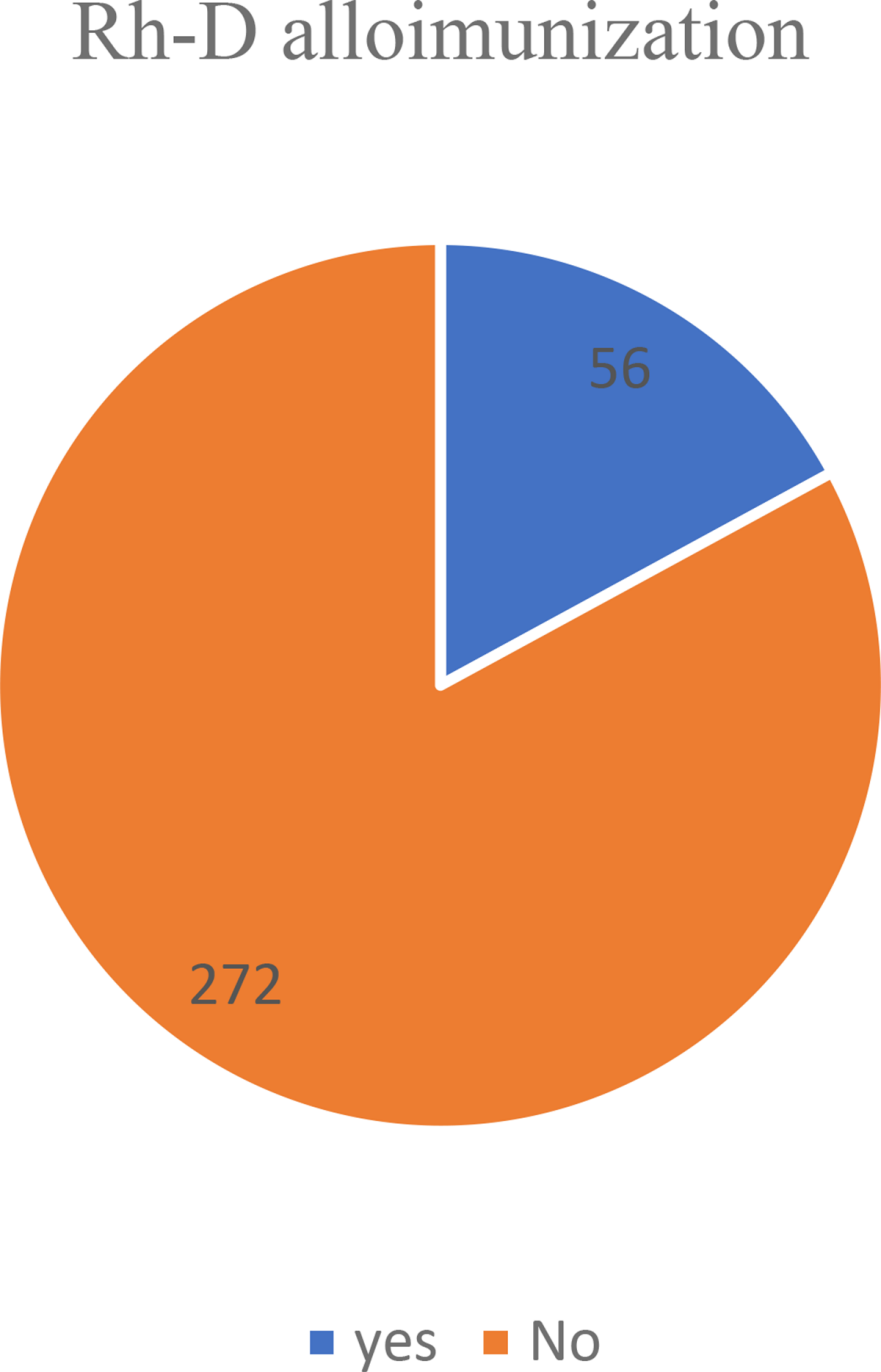
Showing the prevalence of Rh alloimmunization among Rh-negative women in teaching hospitals of Addis Ababa, 2021.

From a total of 15,894 people, 328 (2.1%) (95% CI, 1.56%, and 2.76%) had an Rh-D negative blood type. Among these, 56 (17.1%) with 95% CI (15.1%, 19.23%) were Rh alloimunized (Figure 2).

### Obstetric-related characteristics and reasons for not using anti-D prophylaxis

Among the 328 study participants, 298 (90.9%) reported that they had no antepartum hemorrhage. One-fifth (22.6%) of pregnant women had a caesarean section, while nine (2.7%) had a breech delivery. This study found that five (1.5%) pregnant women underwent an amniocentesis, and 29 (8.8%) had a history of abortion. In terms of prophylaxis, 178 (54.3%) used anti-D prophylaxis, while 150 (45.7%) did not. Among those who did not use prophylaxis, the most common reason was that 47 (14.3%) of pregnant women could not afford it (Table 2).

**Table 2:** Factors aggravating feto-maternal hemorrhage and reasons for not using anti-D among the study participants in TASH, GMH, and ZMH, Addis Ababa, Ethiopia, 2021.

Variables	Categories	Frequency	Percentage
APH	Yes	30	9.1
	No	298	90.9
Caesarean section delivery	Yes	74	22.6
	No	254	77.4
Breech delivery	Yes	9	2.7
	No	319	97.3
Amniocentesis	Yes	5	1.5
	No	323	98.5
Abortion	Yes	29	8.8
	No	299	91.2
Number of pregnancies	1	45	13.7
	2	122	37.2
	3	87	26.5
	≥4	74	22.6
Use of anti-D prophylaxis	Yes	178	54.3
	No	150	45.7
Reason for not using anti-D prophylaxis's	Did not know my blood type	27	8.2
	Health professional did not tell me	41	12.5
	Fail to afford	47	14.3
	Health professional told me not necessary	25	7.6
	Other^a^	10	3.1

^a^ Ectopic pregnancy, External cephalic version.

### Fetal or neonatal complication among Rh D negative pregnant women

In terms of fetal or neonatal complications, nearly one-tenth (9.8%) of Rh-D-negative women had fetal or neonatal complications. The most common complication among those was jaundice (71.8%). In contrast, seventeen (5.2%) pregnant Rh-D-negative women had fetal or neonatal complications during their current pregnancy. Eight (47.1%) of those tested positive for jaundice (Table 3).

**Table 3:** Fetal or neonatal complications among Rh-negative pregnant women in TASH, GMH, and ZMH, Addis Ababa, Ethiopia, 2021.

Variables	Categories	Frequency	Percentage
Fetal/neonatal complications in the previous delivery	Yes	32	9.8
	No	296	90.2
Types of complication	Mental retardation	2	6.3
	Hydrops fetalis	2	6.3
	Jaundice	23	71.8
	Death	3	9.3
	Others^a^	2	6.3
Fetal/neonatal complications in the current pregnancy	Yes	17	5.2
	No	311	94.8
Types of Fetal/neonatal complications in the current pregnancy	Mental retardation	1	5.9
	Hydrops fetalis	1	5.9
	Jaundice	8	47.1
	Death	1	5.9
	Abortion	4	23.5
	Others^b^	2	11.8

^a^ IUFD.

^b^ HDNB.

### Alloimmunization and its associated factors

In the bivariable analysis, alloimmunization was substantially correlated with predictor factors such as occupation, number of pregnancies, the existence of aggravating or sensitizing events, anti-D usage in a previous pregnancy, cesarean delivery, and abortion (Table 4). However, the final model of multivariable logistic regression analysis showed significant associations between alloimmunization and predictor variables such as profession, number of pregnancies, the existence of aggravating or sensitizing events, and anti-D medication in a prior pregnancy.

**Table 4:** Univariate logistic regression analysis of factors associated with Rh alloimmunization among Rh D-negative pregnant women in teaching hospitals of Addis Ababa, Ethiopia, 2021.

Factors	Categories	Rh-D negative		COR (95% CI)
		Non alloimunized	Alloimunized	
Occupation	Employed	148 (54.4%)	18 (58.9%)	1
	Unemployed	124 (45.6%)	38 (41.1%)	2.13 (1.23, 3.54)*
Number of pregnancies	1	39 (16.6%)	6	2.52 (1.37, 4.64)
	2	95 (34.9%)	27 (48.2%)	0.68 (0.32, 1.44)*
	3	70 (25.7%)	17 (30.4%)	0.80 (0.35, 0.81)
	≥4	62 (22.8%)	12 (21.4%)	1
Caesarean section	Yes	59 (21.7%)	15 (26.8%)	0.76 (0.23, 5.43)*
	No	213 (78.3%)	41 (73.2%)	1
Abortion	Yes	24 (8.8%)	5 (8.9%)	1.02 (0.56, 1.23)*
	No	248 (91.2%)	51 (91.1%)	1
Anti-D use in previous pregnancies	Yes	139 (51.1%)	38 (67.9%)	1
	No	133 (48.9%)	18 (32.1%)	2.02 (1.10, 3.71)**
Sensitizing event	Yes	102 (37.5%)	14 (35.7%)	1
	No	170 (62.5%)	42 (64.3%)	0.556(0.289,1.067)***

Key: * = *p*-value < 0.01, ** = *p* < 0.05, *** = *p*-value < 0.001, L & D, labor & delivery; BPCR, birth preparedness and complication readiness.

Accordingly, those study participants who were unemployed were 2.28 times more likely to be sensitized than those who were employed [AOR = 2.28, 95% CI: 1.21, 4.28]. Furthermore, in this study, those study participants who did not received anti-D in a previous pregnancy were 2.08 times more likely to be immunized than their counterparts (AOR = 3.99, 95% CI: 2.20, 7.25). Moreover, this study pointed out that the study participants with an absence of sensitizing events were 48% less likely to develop alloimmunization as compared to their counterparts [AOR = 0.52, 95% CI = 0.27, 0.84] (Table 5).

**Table 5:** Multivariable logistic regression analysis of factors associated with Rh alloimmunization among Rh D-negative pregnant women in teaching hospitals of Addis Ababa, Ethiopia, 2021.

Factors	Categories	Rh D negative		AOR (95% CI)
		Non alloimunized	Alloimunized	
Occupation	Employed	148 (54.4%)	18 (58.9%)	1
	Unemployed	124 (45.6%)	38 (41.1%)	2.28 (1.21, 4.28)*
Number of pregnancies	1	39 (16.6%)	6	2.21 (0.21, 4.28)
	2	95 (34.9%)	27 (48.2%)	0.66 (0.30, 1.43)
	3	70 (25.7%)	17 (30.4%)	0.74 (0.32, 1.71)
	≥4	62 (22.8%)	12 (21.4%)	1
Caesarean section	Yes	59 (21.7%)	15 (26.8%)	1.32 (0.65, 1.34)
	No	213 (78.3%)	41 (73.2%)	1
Abortion	Yes	24 (8.8%)	5 (8.9%)	1.35 (0.89, 1.65)
	No	248 (91.2%)	51 (91.1%)	1
Anti-D use in previous pregnancies	Yes	139 (51.1%)	38 (67.9%)	1
	No	133 (48.9%)	18 (32.1%)	2.08 (1.10, 3.92)**
Sensitizing event	Yes	102 (37.5%)	14 (35.7%)	1
	No	170 (62.5%)	42 (64.3%)	0.52 (0.27, 0.84)***

Key: * = *p*-value < 0.01, ** = *p* < 0.05, *** = *p*-value < 0.001, L & D, labor & delivery; BPCR, birth preparedness and complication readiness.

## Discussion

This study assessed the prevalence and determinants of Rh alloimmunization among Rh-negative pregnant women in the teaching hospital of Addis Ababa, Ethiopia. This study highlighted that of a total of 15,894 people, 328 (2.1%) (95% CI, 1.56%, 2.76%) had an Rh-D-negative blood type. Among them, 56 (17.1%) were Rh-alloimunized. Factors such as occupation, the presence of aggravating or sensitizing events, and the anti-D use of previous pregnancies were identified as predictors of alloimmunization.

In this study, the prevalence of Rh-D negatives and Rh alloimmunization was 2.1% and 17.1%, respectively. The prevalence of RH-D negatives is similar to a study reported from Japan (2.1%) (23), which may be attributable to similar social structures and investigation approaches. Yet, the prevalence of Rh-D-negative is much higher than in previous studies conducted in different settings, like China (0.4%) (24). One of the possible reasons could be related to sampling technique and selection biases. These discrepancies may have an explanation based on variations in sample sizes, evaluation techniques, and study period time gaps. Another argument is that participants in the current study have easier access to information about maternal healthcare. In contrast, this finding was far less significant than those of other investigations, including Arab Minch (6.2%) (20), Jimma (6.3%) (25), Nigeria (9.5%) (26), and western Uganda (3.6%), South Western Uganda (3.9%), and Western Uganda (5.7%) (27–29) respectively.

The socio-demographic characteristics of the study participants, such as their poor socio-economic status and level of education, could be one of the potential causes. Moreover, this suggests that the distribution of Rh-D-negative women varies across Ethiopia. Aside from spatial and ethnic-racial variations, evidence shows that the Rh-D-negative frequency varies temporally in a single population in different regions of the same country (30).

Findings from this study showed that the prevalence of Rh alloimmunization was 17.1%. Results from this study were relatively higher than those from studies conducted in Jimma (14.3%) (31), western Uganda (3.6%), South Western Uganda (3.9%), and Western Uganda (5.7%) (22, 32–34), respectively; a systematic review conducted in sub-Saharan Africa (6.7%) (32), India (4.2%), (6.9%), (10.4%) (22, 33, 34), respectively; and USA (0.74%) (35). The possible justifications include the use of advanced diagnosis and early booking of ANC, sample size, and socioeconomic characteristics of study participants. This may also be related to the fact that clients may fail to receive antibody prophylaxis due to a lack of money to purchase the prophylaxis, exposing pregnant women to sensitization.

In the final model of multivariable analysis, the occupation of study participants was found to be associated with alloimmunization. Thus, those study participants who were unemployed were 2.28 times more likely to be sensitized than those who were employed. The possible justification for this could be that pregnant women who are not employed might be unable to cover the cost of prophylaxis, leaving them vulnerable to alloimmunization. Furthermore, women who were not employed were more likely to have an adverse pregnancy history, such as a miscarriage, stillbirth, or APH, which in turn plays a pivotal role in aggravating a fete maternal hemorrhage, which clearly predisposes pregnant women to alloimmunization (29, 31, 34, 36).

Furthermore, in this study, anti-D use in previous pregnancies was found to be an independent predictor of alloimmunization. Accordingly, those study participants who had not used anti-D in their previous pregnancy were 2.08 times more likely to be immunized than their counterparts. These findings are also supported by studies conducted in Jimma (25), Uganda (27), and India (36). This could be because immunoglobulin prophylaxis plays an important role in the prevention of HDFN and has a significant socioeconomic impact. Immunoglobulin prophylaxis is given to Rh-D-negative mothers twice during pregnancy, once at 28 weeks of gestation and once 72 h after delivery if the fetus is Rh-D positive (37). Moreover, there is a risk of discontinuing regular hospital visits due to a variety of socioeconomic factors. There is always a 10% chance of alloimmunization in such dropout cases due to a lack of immunoglobulin prophylaxis (38).

Finally, the study pointed out that the study participants without sensitizing events were 48% less likely to develop alloimmunization as compared to their counterparts. The findings are also in line with a study conducted in Uganda (27). The possible explanation is that having no sensitizing events results in a reduction in feto-maternal hemorrhage, which is essential to sensitization.

## Limitations of study

It would be hard to establish a causal link between the variable and the analytic result in this study due to the design of the investigation. Further, as there are no advanced laboratory diagnostic tests, we failed to mention the specific types of Rh factors involved except Rh-D antigens. Moreover, to get an estimated sample size, it is explicitly advisable either to include a wide range of health facilities or to extend the duration of the study as we encountered a problem. It is also critical to consider selection bias and add major characteristics such as a lack of information on ANC care and compliance with ANC care, which are both absent. Furthermore, the author strongly suggests doing observational research to vividly describe the association between fetal outcome and Rh alloimmunization.

## Conclusions

This study pointed out that the prevalence of Rh was relatively high, and almost one in every five pregnant women was alloimunized. Factors such as being unemployed and having not used anti-D prophylaxis in previous pregnancies were found to be associated with outcome variables. All stakeholders and concerned entities should pay attention to enhancing access to and affordability of anti-D prophylaxis. Furthermore, standardized and universal prophylaxis administration should be implemented, with the government or relevant entities taking responsibility for covering the costs for those who are unable to do so.

## Data Availability

The original contributions presented in the study are included in the article, further inquiries can be directed to the corresponding author.
